# Prognostic impact of mitochondrial dynamics-related miRNA levels during the treatment of acute heart failure in the hospital

**DOI:** 10.1038/s41598-025-23792-4

**Published:** 2025-11-14

**Authors:** Akihiro Shirakabe, Yoshiyuki Ikeda, Yoshihiro Uchikado, Hirotake Okazaki, Masato Matsushita, Tomofumi Sawatani, Shota Shigihara, Kenichi Tani, Masaki Morooka, Masahito Takahashi, Nobuaki Kobayashi, Mitsuru Ohishi, Junichi Sadoshima, Kuniya Asai

**Affiliations:** 1 Division of Intensive Care Unit, Nippon Medical School Chiba Hokusoh Hospital, 1715 Kamagari, Inzai, Chiba 270-1694 Japan; 2 Department of Cardiovascular Medicine, National Hospital Organization, Minami Kyushu Hospital, Kagoshima, Japan; 3 Department of Cardiovascular Medicine and Hypertension, Graduate School of Medical and Dental Science, Kagoshima University, Kagoshima, Japan; 4 Department of Cell Biology and Molecular Medicine, Rutgers-New Jersey Medical School, Newark, USA; 5 Department of Cardiovascular Medicine, Nippon Medical School, Tokyo, Japan

**Keywords:** Acute decompensated heart failure, Mitochondrial fission, Mitochindrial fusion, Time-dependent changes and Prognosis, Molecular biology, Biomarkers, Cardiology, Diseases

## Abstract

Mitochondrial dynamics-related RNAs during hospitalization for acute heart failure (AHF) were rarely evaluated in various points. In total, 234 patients who visited the emergency room for AHF were retrospectively evaluated. Blood samples were collected within 15 min of admission (day 1), after 48–72 h, and between days 7 and 21. Low miR-140-3p during hospitalization was defined as the level being categorized as Q1 more than once (on days 1, 3 and/or 14), and normal-140-3p during hospitalization as the level never being categorized as Q1. The median miR-140-3p levels were significantly decreased on days 3 and 14 (2.53 [1.06–6.42] and 3.65 [1.41–9.05], respectively) in comparison to the value on day 1 (6.71 [2.66–14.0]). Kaplan–Meier curves indicated that all-cause mortality within 1000 days was significantly higher in the low-miR-140-3p group than in the other-miR-140-3p group on days 1 and 14. Moreover, the survival rate was significantly lower and the rate of HF events was significantly higher in the low-miR-140-3p group than in the normal-miR-140-3p group. The miRNA levels of patients further decreased during treatment for AHF. Low levels of mitochondrial fission-related miRNAs during AHF treatment were independently associated with an increased risk of long-term adverse outcomes.

## Introduction

Circulating miRNAs that regulate mitochondrial dynamics have previously been reported in the field of cardiovascular medicine. In cardiac hypertrophy, miR-485-5p was shown to inhibit mitochondrial fission by targeting mitochondrial-anchored protein ligase and increasing levels of the fusion factor mitofusin (Mfn)2^1^. MiR-30 induces mitochondrial fusion and helps facilitate the delivery of mesenchymal stromal cells via extracellular vesicles. Consequently, miR-30 contributes to the improvement of acute kidney ischemia–reperfusion injury by inhibiting excessive mitochondrial fission^2^. Although mitochondrial dynamics-related miRNAs have rarely been reported in patients with cardiovascular disease, we recently conducted clinical research on mitochondria-related miRNAs in patients with compensated heart failure (HF)^3^. Circulating levels of miR-140-5p were independently associated with the presence of mitochondrial fission, and a significant correlation was noted between miR-140-5p levels and mitochondrial area.

We also evaluated the role of miRNAs in patients with acute HF (AHF). Circulating miRNAs, particularly miR-140, were decreased in patients with AHF, suggesting that mitochondrial fission is severely impaired in the hyperacute phase of the condition. Cardiac biomarkers were elevated in patients with AHF with reduced miR-140 levels, and the prognostic impact of decreased circulating miR-140 levels was noted in the AHF cohort. However, evaluation of various sampling points in miRNAs during treatment for AHF remain unknown. The hemodynamic status of a patient with AHF can change drastically within a short period of time. Therefore, measurement of miRNAs and evaluation of the prognostic value of miRNAs during treatment for AHF in hospital may be important. We hypothesized that miRNA levels, not only upon admission but also during treatment, may have important prognostic value. In the present study, we first investigated the miRNAs in various sampling points during hospitalization for AHF and subsequently evaluated the prognostic impact of miRNAs in an AHF cohort.

## Methods

### Subjects

We enrolled 234 consecutive patients who presented to the emergency room of Nippon Medical School, Chiba Hokusoh Hospital, with AHF from December 2016 to December 2018 and in whom miRNAs (miR-30-3p, miR-140-3p and miR-485-3p) were collected within 15 min of visiting the emergency room and 3 days after admission. The miRNAs were measured retrospectively using residual serum samples of previous prospective study.^4^ Among these, 56 patients either died within 14 days or had no samples collected on day 14. As a result, we had data for day 14 for 178 patients with AHF. Admission day was selected as the timing before treatment, 3 days after admission was as the timing after treatment in intensive care unit (ICU), and 14 days after admission was after treatment in general word, thus before discharge.

AHF is defined as a gradual or rapid change in HF signs and symptoms necessitating urgent therapy. HF was diagnosed by the treating physician at the outpatient clinic according to the European Society of Cardiology (ESC) and Japanese Circulation Society guidelines^5,6^. The physicians first considered the patients’ clinical history (i.e., symptoms, functional limitation, prior cardiac disease, risk factors, exacerbating factors, comorbidities, and drugs used), physical examination results (i.e., vital signs, weight and volume of the heart, lungs, abdomen, and peripheral vascular regions), and results of initial investigations (i.e., chest radiography, 12-lead electrocardiography, and laboratory measurements of troponins, blood urea nitrogen [BUN], creatinine, sodium, potassium, glucose, liver function, and complete blood count). Furthermore, plasma natriuretic peptide was evaluated and echocardiography performed to verify the diagnosis of HF. Echocardiography was used to assess the left/right ventricular systolic and diastolic function and ventricular/atrial volume based on the ESC guideline. All enrolled patients were diagnosed with AHF (either new-onset or decompensated chronic HF with symptoms sufficient to warrant hospitalization) in the emergency department according to the aforementioned procedure within 30 min of admission by the treating physician.

Patients requiring any of the following were admitted to the ICU: (1) high-concentration oxygen inhalation (including mechanical support) for orthopnea; (2) inotropic or mechanical support for low blood pressure; or (3) various types of diuretics for general or lung edema. Other patients were admitted to general wards. The treatment strategy was left to the discretion of each physician. In all cases, diuretics or vasodilators were administered for AHF.

### Measurement of circulating miRNAs

MiR-30 and miR-485 reportedly induce mitochondrial fusion, and miR-140 reportedly causes mitochondrial fission^1,2,7^. Therefore, we measured the levels of miR-30-3p, miR-485-3p, and miR-140-3p in our study. Levels of mitochondrial dynamics-related miRNAs were measured via quantitative reverse transcription polymerase chain reaction (PCR) according to a published protocol^8^. Briefly, total RNA was isolated from serum samples using the miRNeasy Serum/Plasma kit (Qiagen, Germantown, MD, USA), followed by reverse transcription into cDNA and quantitative PCR using the TaqMan Advanced miRNA Assays Kit (Thermo Fisher Scientific, Waltham, MA, USA). The levels of these miRNAs were measured in duplicate and normalized to those of cel-miR-39-3p. Primers for PCR were purchased from Invitrogen, Thermo Fisher Scientific. The miRNA sequences and their miRBase accession numbers are shown in Table 1.

**Table 1:** miRNA sequences.

miRNA	Primer sequence	miRBase accession number
miR-30-3p	CUUUCAGUCGGAUGUUUGCAGC	MIMAT0000088
miR-30-5p	UGUAAACAUCCUCGACUGGAAG	MIMAT0000087
miR-140-3p	UACCACAGGGUAGAACCACGG	MIMAT0004597
miR-140-5p	CAGUGGUUUUACCCUAUGGUAG	MIMAT0000431
miR-485-3p	GUCAUACACGGCUCUCCUCUCU	MIMAT0002176
miR-485-5p	AGAGGCUGGCCGUGAUGAAUUC	MIMAT0002175
cel-miR-39-3p	UCACCGGGUGUAAAUCAGCUUG	MIMAT0000010

### Procedure

We provide the sub-group analysis using mi-140-3p to investigate the mitochondrial fission during the treatment of AHF. In our previous clinical research on mitochondria-related miRNAs, the miR-140-5p levels were significant correlated with mitochondrial area, and circulating levels of miR-140-5p were independently associated with the presence of mitochondrial fission in patients with HF. We therefore decided to perform subgroup analysis using miR-140-3p for evaluating mitochondrial fission during treatment of AHF.

The miR-140-3p value were undetermined in four patients on days 3 and in five patients on days 14. The AHF cohort was divided into two groups according to the quartiles of miR-140-3p on days 3 and 14: low (Q1, miR-140-3p < 1.05, n = 58) and other (Q2, Q3, and Q4, miR-140-3p ≥ 1.05, n = 172) on day 3 and low (Q1, miR-140-3p < 1.40, n = 43) and other (Q4, miR-140-3p ≥ 1.40, n = 130) on day 14.

Subsequently, subgroup analyses were performed for the low- and normal-miR-140-3p groups after treatment for AHF according to the following definitions: low miR-140-3p during hospitalization was defined as being categorized in Q1 more than once during days 1, 3, and/or 14, and normal-140-3p during hospitalization was defined as never being categorized in Q1.

The patients’ characteristics, including age, sex, presence of de novo or recurrent HF, etiology of HF, risk factors for atherosclerosis (diabetes mellitus, hypertension, and dyslipidemia), vital signs (systolic blood pressure [SBP] and heart rate), left ventricular ejection fraction (LVEF) upon echocardiography, presence of orthopnea and chronic kidney disease, prescription of hemodialysis, arterial blood-gas data, laboratory data (e.g., BUN, creatinine, estimated glomerular filtration rate, total bilirubin, hemoglobin, brain natriuretic peptide [BNP], and C-reactive protein [CRP]), mechanical support (non-invasive positive-pressure ventilation [NPPV] and endotracheal intubation), medications administered during ICU admission, and duration of admission (duration of ICU stay and hospital stay), were compared between the low- and other-miR-140-3p groups on day 3, and day 14, and between the low-miR-140-3p and normal-miR-140-3p during the entire hospitalization period.

The LVEF was calculated at admission using the Teichholz method or Simpson’s method, the method being decided on a case-by-case basis (Sonos 5500; Hewlett Packard, Palo Alto, CA, USA; or Vivid I; GE Yokogawa Medical, Tokyo, Japan). As the LVEF was measured during the acute phase, the measurement was inadequate for patients with severe orthopnea.

### Short- and long-term prognoses

Short-term outcomes were evaluated as the duration of hospital stay and in-hospital mortality. The long-term outcome were all-cause mortality and HF events within 1000 days. An HF event was defined as all-cause mortality or readmission for HF. Patients underwent clinical follow-up via routine outpatient visits. The outcomes of patients who were monitored at other institutions were determined via telephone. The prognostic value of miR-140-3p levels, in terms of 1000-day all-cause death, was evaluated using a multivariable logistic regression model and Kaplan–Meier-curve analysis. A log-rank test was performed to determine the significance of differences.

### Statistical analyses

All of the data were statistically analyzed using the SPSS 22.0J software program (SPSS Japan Institute, Tokyo, Japan). Numerical data were expressed as the median and the 25th to 75th percentiles. The Mann–Whitney U test was used to compare two groups. The chi-squared test was used for comparisons of proportions. Time-dependent differences on days 1, 3, and 14 were evaluated using a one-way analysis of variance (ANOVA). P-values < 0.05 were considered to indicate statistical significance.

The prognostic value of the miR-140-3p level on days 1, 3, and 14, including that in the subgroups during hospitalization, was assessed using a Cox proportional-hazards regression model. Cox regression analysis was also performed to determine the hazard ratio (HR) for all-cause mortality and HF events. All clinically relevant factors affecting the prognosis, including age ≥ 60 years, sex (male), SBP (< 100 mmHg), heart rate (per 10-beats/min increase), sodium (per 1.0-mEq/L increase), creatinine (per 0.1-mg/dL increase), total bilirubin (per 1.0-mg/dL increase), and LVEF (< 40%), were included in the multivariable Cox proportional-hazards model to identify factors associated with all-cause mortality and HF events. Included number of variables in the Cox regression analysis were decided by one-eighth to one-tenth for total event number. These factors were included in the model using simultaneous forced entry. The cumulative survival rates and HF events in each of the two groups were analyzed using Kaplan–Meier curves, and the log-rank test was used to calculate the statistical significance of the differences.

### Ethical considerations

The Research Ethics Committee of Nippon Medical School, Chiba Hokusoh Hospital, approved the study protocol. Written informed consent was obtained from all participants before commencing the study. Due to its retrospective design for the evaluation of miRNAs, the need for written informed consent was waived. Based on the advice of the Ethics Committee, the present study was presented as a poster displayed at the hospital and published on the hospital’s homepage, where it could be viewed by anyone. All procedures were performed in accordance with the tenets of the Declaration of Helsinki.

## Results

### Time-dependent changes in miRNAs and differences in patient characteristics and outcomes

**Fig. 1:**
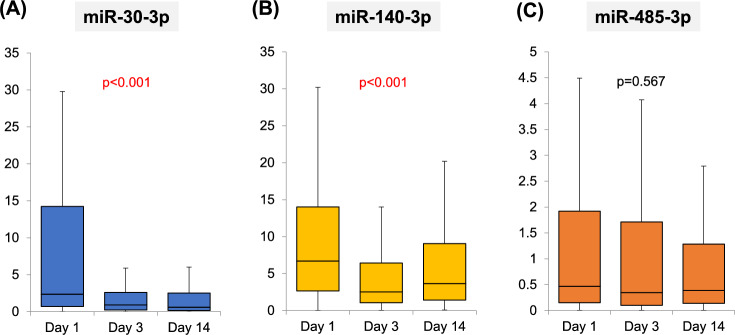
Time-dependent changes in miRNA levels during treatment for AHF. The mi-30p-3p and mi-140-3p was significantly decreased on day 3 in comparison with that on day 1, after which the level plateaued. Values are expressed as the median and the 25th to 75th percentiles. AHF, acute heart failure; day 1, within 15 min of admission; day 3, on hospital day 3 (after 48–72 h); day 14, on hospital day 14 (between days 7 and 21).

The miR-30-3p levels were significantly decreased on days 3 and 14 (0.93 [0.22–2.60] and 0.60 [0.16–2.52] pmol/h/mL, respectively) in comparison to the value on day 1 (2.35 [0.71–14.2] pmol/h/mL). The same was true for the miR-140-3p level (2.53 [1.06–6.42] and 3.65 [1.41–9.05], respectively, vs. 6.71 [2.66–14.0]) (Fig. 1).

The AHF patient cohort consisted of 152 (65.0%) male patients, and the median age was 76 years. A total of 153 (65.4%) patients had new-onset HF, and 107 (45.7%) had ischemic heart disease. The majority of patients (70.5%) had orthopnea upon admission, and the median LVEF upon admission was 35.0% (Table 2). Diabetes mellitus was significantly more common in the low-miR-140-3p group than in the other-miR-140-3p group on day 3. In the low-miR-140-3p group, the hemoglobin levels were significantly lower, whereas the serum BUN and creatinine levels were non-significantly higher than in the other-miR-140-3p group on day 3 (Table 2). Similar observations were made on day 14; diabetes mellitus was significantly more common, and significantly more endotracheal intubations were performed in the low-miR-140-3p group than in the other-miR-140-3p group. In the low-miR-140-3p group, the serum BUN was significantly higher and creatinine levels were non-significantly higher than in the other-miR-140-3p group on day 14. Notably, serum N-terminal-proBNP, heart-type fatty acid binding protein (HFABP), and high-sensitivity troponin T (hsTnT) levels were significantly higher in the low-miR-140-3p group (11,193 [2873–21682] pg/mL, 19.4 [10.9–48.9] mg/mL, and 0.09 [0.05–0.36] ng/mL, respectively) than in the other-miR140-3p group (5509 [2564–12770] pg/mL, 12.6 [6.4–25.5] mg/mL, and 0.06 [0.03–0.14] ng/mL, respectively) on day 3, and serum BNP and NT-pro BNP levels were significantly higher in the low-miR-140-3p group (1165 [617–1841] pg/ml and 10,305 [5054–23183] pg/mL, respectively) than in the other-miR140-3p group (793 [458–1219] pg/mL and 6401 [2538–12919] pg/mL, respectively) on day 14.

**Table 2:** Patient characteristics in groups separated according to miR-140-3p quartiles.

	All (N = 234)	Day 3 (n = 230)		p value	Day 14 (n = 173)		p value
		Low: Q1	Others: Q2/Q3/Q4		Low: Q1	Others: Q2/Q3/Q4	
		miR-140-3p < 1.05	miR-140-3p ≥ 1.05		miR-140-3p < 1.40	miR-140-3p ≥ 1.40	
		(n = 58)	(n = 172)		(n = 43)	(n = 130)	
Characteristics							
Age (years)	76 (67–82)	77 (67–84)	75 (67–82)	0.416	78 (68–84)	74 (65–83)	0.392
Type (readmission, %)	81 (34.6%)	18 (31.0%)	61 (35.5%)	0.632	21 (48.8%)	43 (33.1%)	0.071
Sex (male, %)	152 (65.0%)	34 (58.6%)	116 (67.4%)	0.265	25 (58.2%)	83 (63.8%)	0.586
Etiology (ischemia, %)	107 (45.7%)	30 (51.7%)	76 (43.6%)	0.291	22 (51.2%)	54 (41.5%)	0.292
Risk factors for atherosclerosis							
Hypertension (yes, %)	181 (77.3%)	48 (82.8%)	129 (75.0%)	0.28	33 (76.7%)	98 (75.4%)	1
Diabetes mellitus (yes, %)	105 (44.9%)	34 (58.6%)	70 (40.7%)	**0.022**	27 (62.8%)	48 (36.9%)	**0.004**
Dyslipidemia (yes, %)	126 (53.8%)	33 (56.9%)	92 (53.5%)	0.761	27 (62.8%)	69 (53.1%)	0.293
Vital signs and status							
Systolic blood pressure (mmHg)	153 (125–179)	148 (110–171)	154 (128–183)	0.111	137 (119–171)	152 (126–179)	0.311
Heart rate (beats/min)	100 (85–117)	98 (84–111)	103 (86–120)	0.158	100 (90–111)	100 (84–119)	0.839
LVEF (%)	35 (26–50)	41 (29–56)	34 (25–47)	**0.048**	32 (23–44)	35 (24–50)	0.245
Orthopnea (yes, %)	165 (70.5%)	40 (69.0%)	123 (71.5%)	0.74	34 (79.1%)	91 (70.0%)	0.326
Respiratory management							
ETI (yes, %)	17 (7.3%)	7 (12.1%)	10 (5.8%)	0.145	6 (14.0%)	5 (3.8%)	**0.029**
NPPV (yes, %)	139 (59.4%)	29 (50.0%)	109 (63.4%)	0.088	29 (67.4%)	75 (57.7%)	0.285
Arterial blood gas							
pH	7.38 (7.29–7.44)	7.39 (7.29–7.46)	7.38 (7.29–7.44)	0.723	7.37 (7.27–7.45)	7.39 (7.32–7.45)	0.313
PCO_2_ (mmHg)	37 (32–48)	36 (30–49)	38 (33–48)	0.309	38 (33–49)	36 (31–45)	0.216
PO_2_ (mmHg)	100 (77–148)	102 (77–146)	99 (78–145)	0.962	99 (73–139)	106 (80–154)	0.339
HCO_3_^-^ (mmol/L)	21.8 (19.2–25.3)	21.7 (18.6–25.1)	21.8 (19.5–25.4)	0.589	21.5 (18.8–25.3)	22.0 (19.2–25.4)	0.964
SaO_2_ (%)	97 (94–99)	97 (95–99)	97 (94–99)	0.634	97 (93–99)	98 (95–99)	0.197
Lactate (mmol/L)	1.7 (1.1–3.3)	1.8 (1.1–3.2)	1.7 (1.1–3.1)	0.676	1.8 (1.3–3.1)	1.8 (1.0–3.4)	0.651
Laboratory data							
WBC (mg/dL)	9000 (6700–11,900)	10,270 (6793–12,953)	8805 (6660–11,825)	0.265	8310 (7215–11,850)	9220 (6678–11,850)	0.826
Total bilirubin (mg/dL)	0.7 (0.5–1.1)	0.6 (0.3–1.2)	0.7 (0.5–1.0)	0.671	0.7 (0.4–1.1)	0.6 (0.5–1.1)	0.783
BUN (mg/dL)	25.0 (18.1–41.0)	28.7 (20.5–48.6)	24.2 (17.8–37.3)	0.074	29.9 (21.2–47.5)	23.4 (18.0–36.0)	**0.041**
Creatinine (mg/dL)	1.21 (0.87–2.03)	1.34 (0.97–2.57)	1.19 (0.84–1.85)	0.075	1.54 (1.01–2.31)	1.18 (0.85–1.64)	0.051
Sodium (mmol/L)	140 (137–143)	139 (136–144)	140 (137–143)	0.389	139 (136–143)	140 (137–143)	0.509
Potassium (mmol/L)	4.3 (3.9–4.7)	4.4 (3.9–4.8)	4.3 (3.9–4.6)	0.161	4.2 (3.9–4.7)	4.3 (3.9–4.7)	0.91
Uric acid (mg/dL)	6.8 (5.5–7.9)	6.3 (5.0–7.2)	6.8 (5.6–8.3)	**0.016**	6.8 (5.2–7.6)	6.8 (5.7–7.8)	0.359
Hemoglobin (g/dL)	12.1 (10.2–13.9)	10.9 (9.9–12.4)	12.5 (10.5–14.4)	**0.002**	11.5 (10.4–13.2)	12.3 (10.0–14.1)	0.31
CRP (mg/dL)	0.97 (0.22–3.56)	1.44 (0.32–6.01)	0.85 (0.22–2.95)	0.052	1.36 (0.32–4.40)	0.74 (0.22–3.59)	0.257
Cardiac biomarkers							
BNP (pg/mL)	837 (459–1462)	847 (414–1678)	826 (482–1330)	0.507	1165 (617–1841)	793 (458–1219)	**0.015**
NT-proBNP (pg/mL)	6541 (2735–14,215)	11,193 (2873–21,682)	5509 (2564–12,770)	**0.016**	10,305 (5054–23,183)	6401 (2538–12,919)	**0.007**
HFABP (mg/mL)	14.3 (6.8–30.6)	19.4 (10.9–48.9)	12.6 (6.4–25.5)	**0.008**	19.4 (10.0–39.2)	13.1 (6.8–24.4)	0.06
hsTnT (ng/mL)	0.06 (0.03–0.17)	0.09 (0.05–0.36)	0.06 (0.03–0.14)	**0.004**	0.07 (0.05–0.17)	0.06 (0.03–0.15)	0.324
Short-term outcome							
Total hospitalization (days)	26 (18–40)	33 (21–47)	23 (16–37)	**0.008**	36 (22–59)	27 (19–37)	**0.001**
In-hospital mortality (yes, %)	23 (9.8%)	9 (15.5%)	14 (8.2%)	0.128	7 (16.3%)	8 (6.2%)	0.058

BNP, brain natriuretic peptide; BUN, blood urea nitrogen; CRP, C-reactive protein; ETI, endotracheal intubation; HFABP, heart-type fatty acid binding protein; hsTnT; high-sensitivity troponin T; LVEF, left ventricular ejection fraction measured using echocardiography; NPPV, non-invasive positive pressure ventilation; NT-proBNP, N-terminal pro-brain natriuretic peptide; PCO_2_, partial pressure of carbon dioxide; PO_2_, partial pressure of oxygen; SaO_2_, oxygen saturation; WBC, white blood P-values were determined using the Mann–Whitney U test/Kruskal–Wallis test or the χ^2^ test.

All numerical data were expressed as medians (25th to 75th percentiles).

Significant values are in bold.

**Fig. 2:**
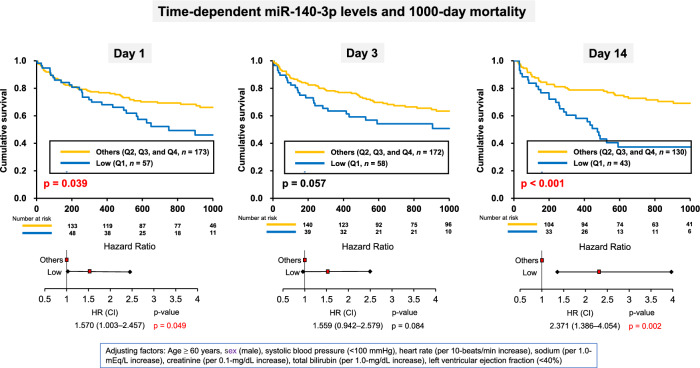
Kaplan–Meier survival curves for each sampling point. (**A**) The outcome, all-cause mortality, in the low-miR-140-3p group was significantly more frequent in comparison to that in the other-miR-140-3p group. (**B**) The outcome, all-cause mortality, was non-significantly more frequent in the low-miR-140-3p group than in the other-miR-140-3p group. (**C**) The outcome, all-cause mortality, in the low-miR-140-3p group was significantly more frequent in comparison with the other-miR-140-3p group.

The Kaplan–Meier survival curves for all-cause mortality within 1000 days, according to miR-140-3p level upon admission and on days 3 and 14, are shown in Fig. 2. Total all cause death and HF events during 1000 days were high with 81 (34.6%) and 114 (48.7%), respectively, in total cohort. Survival rates were significantly lower in the low-miR-140-3p group than in the other-miR-140-3p group upon admission and on day 14, and non-significantly lower on day 3. The multivariable Cox regression model revealed that low-miR-140-3p levels upon admission and on day 14 were independent predictors of 1000-day mortality (HR: 1.570, 95% confidence interval [CI] 1.003–2.457, p = 0.049 and HR: 2.371, 95% CI 1.386–4.054, p = 0.002, respectively) (Fig. 2).

### Differences in patient characteristics and outcomes during hospitalization

Diabetes mellitus and orthopnea were significantly more frequent in the low-miR-140-3p group than in the normal-miR-140-3p group. In the low-miR-140-3p group, hemoglobin levels were significantly lower, whereas the serum BUN, creatinine, and CRP levels were significantly higher, in comparison with those in the normal-miR-140-3p group (Table 3). Serum BNP, NT-pro BNP, HFABP, and hsTnT levels were significantly higher in the low-miR-140-3p group (1000 [590–1779] pg/mL, 10,170 [4560–23387] pg/mL, 19.4 [9.2–42.3] mg/mL, and 0.08 [0.04–0.20] ng/mL, respectively) than in the normal-miR140-3p group (756 [450–1106] pg/mL, 4179 [2096–10370] pg/mL, 10.3 [5.9–20.0] mg/mL, and 0.05 [0.03–0.11] ng/mL, respectively).

**Table 3:** Patient characteristics in groups separated according to miR-140-3p levels.

	During hospitalization (N = 234)		p value
	Low	Normal	
	(n = 118)	(n = 116)	
Characteristics			
Age (years)	75 (67–83)	76 (67–82)	0.856
Type (readmission, %)	45 (38.1%)	36 (31.0%)	0.274
Sex (male, %)	71 (60.2%)	81 (69.8%)	0.133
Etiology (ischemia, %)	55 (46.6%)	52 (44.8%)	0.794
Risk factors for atherosclerosis			
Hypertension (yes, %)	92 (78.0%)	89 (76.7%)	0.876
Diabetes mellitus (yes, %)	65 (55.1%)	40 (34.5%)	**0.002**
Dyslipidemia (yes, %)	68 (57.6%)	58 (50.0%)	0.294
Vital signs and status			
Systolic blood pressure (mmHg)	150 (120–174)	154 (130–183)	0.182
Heart rate (beats/min)	99 (84–113)	108 (87–125)	0.144
LVEF (%)	38 (23–50)	34 (27–50)	0.987
Orthopnea (yes, %)	91 (77.1%)	74 (63.8%)	**0.031**
Respiratory management			
ETI (yes, %)	12 (10.2%)	5 (4.3%)	0.129
NPPV (yes, %)	74 (62.7%)	65 (56.0%)	0.352
Arterial blood gas			
pH	7.38 (7.29–7.44)	7.39 (7.30–7.44)	0.506
PCO_2_ (mmHg)	37 (30–49)	38 (33–47)	0.512
PO_2_ (mmHg)	99 (75–148)	103 (79–147)	0.729
HCO_3_^-^ (mmol/L)	21.6 (18.6–24.9)	22.3 (19.9–25.8)	0.138
SaO_2_ (%)	98 (94–99)	97 (95–99)	0.875
Lactate (mmol/L)	1.8 (1.1–3.1)	1.6 (1.1–3.1)	0.589
Laboratory data			
WBC (mg/dL)	8945 (6700–11,700)	9035 (6730–11,918)	0.876
Total bilirubin (mg/dL)	0.7 (0.4–1.1)	0.6 (0.5–1.0)	0.796
BUN (mg/dL)	30.6 (20.7–48.3)	21.5 (17.4–30.0)	** < 0.001**
Creatinine (mg/dL)	1.44 (0.97–2.45)	1.06 (0.83–1.52)	** < 0.001**
Sodium (mmol/L)	139 (137–143)	141 (137–143)	0.155
Potassium (mmol/L)	4.4 (3.9–4.8)	4.3 (3.8–4.6)	**0.043**
Uric acid (mg/dL)	6.7 (5.2–7.7)	6.8 (5.7–8.3)	0.221
Hemoglobin (g/dL)	11.0 (9.7–12.7)	12.9 (10.7–15.1)	** < 0.001**
CRP (mg/dL)	1.50 (0.30–4.92)	0.66 (0.20–1.99)	**0.004**
Cardiac biomarkers			
BNP (pg/mL)	1000 (590–1779)	756 (450–1106)	**0.005**
NT-proBNP (pg/mL)	10,170 (4560–23,387)	4179 (2096–10,370)	** < 0.001**
HFABP (mg/mL)	19.4 (9.2–42.3)	10.3 (5.9–20.0)	** < 0.001**
hsTnT (ng/mL)	0.08 (0.04–0.20)	0.05 (0.03–0.11)	**0.001**
Short-term outcome			
Total hospitalization (days)	30 (20–46)	22 (15–35)	**0.005**
In-hospital mortality (yes, %)	16 (13.6%)	7 (6.0%)	0.077

BNP, brain natriuretic peptide; BUN, blood urea nitrogen; CRP, C-reactive protein; ETI, endotracheal intubation; HFABP, heart-type fatty acid binding protein; hsTnT; high-sensitivity troponin T; LVEF, left ventricular ejection fraction measured using echocardiography; NPPV, non-invasive positive pressure ventilation; NT-proBNP, N-terminal pro-brain natriuretic peptide; PCO_2_, partial pressure of carbon dioxide; PO_2_, partial pressure of oxygen; SaO_2_, oxygen saturation; WBC, white blood cells.

P-values were determined using the Mann–Whitney U test/Kruskal–Wallis test or the χ^2^ test.

All numerical data were expressed as medians (25th to 75th percentiles).

Significant values are in bold.

**Fig. 3:**
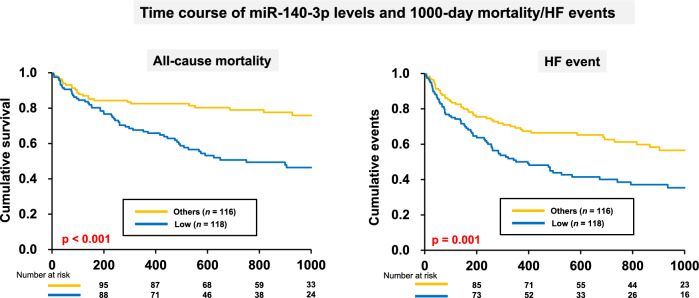
Kaplan–Meier survival curves in the low- and normal-miR-140-3p groups during hospitalization. (**A**) The outcome, all-cause mortality, was significantly more frequent in the low-miR-140-3p group than in the normal-miR-140-3p group. (**B**) The outcome, HF events, was significantly more common in the low-miR-140-3p group than in the normal-miR-140-3p group. HF, heart failure.

The Kaplan–Meier survival curves for all-cause mortality and HF events within 1000 days, according to miR-140-3p level during hospitalization, are shown in Fig. 3. Survival plus event-free rates were significantly lower in the low-miR-140-3p group than in the normal-miR-140-3p group (Fig. 3). The multivariable Cox regression model revealed that low miR-140-3p levels upon admission and on day 14 were independent predictors of 1000-day mortality and HF events (HR: 2.566, 95% CI 1.578–4.173, p < 0.001 and HR: 1.902, 95% CI 1.287–2.811, p = 0.001, respectively) (Table 4).

**Table 4:** Multivariate Cox regression analysis for incidence of 1000-day mortality and HF events.

	Time-dependent groups					
	All-cause mortality			HF events		
	HR	95% CI	p	HR	95% CI	p
miR-140-3p < 2.75	2.566	1.578–4.173	** < 0.001**	1.902	1.287–2.811	**0.001**
Age ≥ 60 years old	3.819	1.489–9.796	**0.005**	1.889	1.003–1.359	**0.049**
Sex (male)	0.848	0.522–1.378	0.505	0.901	0.597–1.359	0.619
SBP < 100 mmHg	2.878	1.568–5.285	** < 0.001**	2.04	1.161–3.854	**0.013**
Heart rate (per 10-beats/min increase)	0.993	0.918–1.074	0.858	1.012	0.947–1.082	0.72
Total bilirubin (per 0.1-mg/dL increase)	0.993	0.965–1.022	0.624	0.985	0.959–1.012	0.28
Creatinine (per 0.1-mg/dL increase)	1.002	0.992–1.013	0.674	1	0.991–1.009	0.941
Sodium (per 1.0-mEq/L increase)	0.974	0.928–1.023	0.29	0.98	0.941–1.021	0.34
LVEF < 40%	1.759	1.051–2.945	**0.032**	1.859	1.215–2.844	**0.004**

HF, heart failure; HR, hazard ratio; CI, confidence interval; SBP, systolic blood pressure.

LVEF, left ventricular ejection fraction.

Significant values are in bold.

## Discussion

In this study, miR-140-3p and miR-30-3p levels continued to decrease during in-hospital treatment for AHF. Low miR-140-3p levels upon admission and 14 days after admission were independently risk factors for mortality within 1000 days. Being categorized in the low-miR-140-3p group more than once during hospitalization was also an independent risk factor for both all-cause mortality and HF events within 1000 days. These results underscore the prognostic importance of severe suppression of mitochondrial fission during treatment for AHF. Although mitochondrial dynamics were reported to be associated with cardiac dysfunction in some previous study, it was using the cultured myocyte and/or animals.^9^ As it had been mainly evidenced by basal research, the present study which was evaluated by human blood samples has novelty.

Mitochondria are normally regulated by quality control mechanisms, including fission and fusion, and autophagy ^10,11^. Impairment of any of these mitochondrial quality control processes leads to mitochondrial dysfunction and cell death. Drp1 is a small GTPase that mediates mitochondrial fission, and plays an important role in mitochondrial autophagy in cardiomyocytes at baseline and in response to energy stress ^10^. Although Drp1 is localized primarily in the cytosol, it is recruited to mitochondria in response to stress, thereby inducing mitochondrial fission and/or mitochondrial autophagy in the heart ^10^. Constitutive inhibition of mitochondrial fission by Drp1 knockdown facilitated cardiac damage induced either by I/R or pressure overload due to the incompetency of mitochondria-targeted autophagy^10,12^. Meanwhile, it has been shown that cardiac and mitochondrial dysfunction caused by downregulation of Miff can be rescued by concomitant downregulation of Mfn1^13^, suggesting that balancing mitochondrial fusion and fission is critical for the maintenance of mitochondrial function in the heart. Aside from the effect on mitochondrial autophagy, whether or not the lack of appropriate fission in Drp1-hetCKO mice also contributes to the enhancement of HF in mice during PO remains to be elucidated^12^. From these basal perspectives, excessive suppression of mitochondrial fission would be presumed to be detrimental in heart.

Since it is impossible to evaluate the mitochondrial dynamics in heart during clinical emergent situation, we reported our approach to predicting the mitochondrial dynamics by using mitochondrial dynamics-related RNAs^8^. Circulating miR-140 levels reportedly play a pivotal role in the promotion of fission and cardiomyocyte apoptosis via negative regulation of Mfn1 in previous basal research.^7^ MiR-140 is integrated into the mitochondrial network through its regulation of Mfn1. In clinical research, plasma miR-140-3p levels were elevated in patients with ACS compared with those in patients with stable angina or healthy subjects ^14^. Another clinical study showed that circulating miR-140-3p predicted cardiovascular death in ACS^15^. We recently reported in our conducted clinical research that circulating levels of miR-140-5p were independently associated with the presence of mitochondrial fission, and a significant correlation was noted between miR-140-5p levels and mitochondrial area by endomyocardial biopsy of HF patients^3^. As mitochondrial dynamics-related miRNAs have rarely been reported in patients with HF, our recent conducted this clinical research was key for our present study. Our effort to know the mitochondria dynamics by using miRNA in AHF clinical setting has therefore novelty.

The number of AHF patients has increased in Japan during the “HF pandemic” era^16,17^, and is becoming an increasingly serious concern as the Japanese and global population ages. AHF is fundamentally recognized as a heterogeneous condition that may have many comorbidities, including malnutrition, cachexia, anemia, infection, sarcopenia, mental disorder, and frailty. Various approaches to predicting the adverse events and outcomes of AHF^18–21^ and epidemiological issues^22–26^ have been suggested recently; however, the clinical approaches to know mitochondrial dynamics in cardiomyocytes has never been reported in patients with AHF.

We first demonstrated that impaired miR-140 expression in the emergency-care setting is an important long-term prognosticator. A non-invasive, straightforward, and highly specific blood test for prognostic evaluation in patients with AHF is urgently needed in emergency-care settings. Circulating miRNAs hold potential as such practical prognostic tools. We can successfully evaluate the mitochondrial dynamics by using human blood sample in AHF clinical emergent setting. It is the first trial evaluating the mitochondria dynamics of human heat in AHF.

In our study, reduced miR-140-3p levels that remained or developed during hospitalization were indicative of adverse outcomes of AHF treatment. The time-dependent changes in miR-140-3p levels also had significant impacts on patient outcomes. Although AHF is recognized as a fundamentally heterogeneous condition with many potential comorbidities, its management in the emergency room is largely uniform, according to guidelines^6^. An appropriate clinical approach for the treatment of patients with vascular failure has been established; the earlier initiation of NPPV and the administration of vasodilators to reduce the afterload are considered important aspects of the initial management of patients who visit the emergency department.^5,27,28^ As a result, most patients with vascular failure recover within days, explaining why their ICU stay is shorter, and the prognosis better, than in other patients with AHF. Thus, mitochondrial quality control will recover via appropriate clinical management in patients with these vascular failure patients. Abnormal mitochondrial dynamics are well known to be involved in mitochondrial dysfunction and result in cell death. The disequilibrium in such dynamics is also a causative factor of myocardial hypoxia. Patients in whom AHF is not easily compensated remain in a state of cardiac overload for several days, during which their cardiomyocytes experience high levels of physical stress. This state causes or maintains impairments in mitochondrial quality control in cardiomyocytes, resulting in cell death and/or myocardial hypoxia, ultimately contributing to long-term adverse outcomes. Although the decrease in miRNA levels varied among patients, continuing and/or newly developed impairment of miR-140-3p levels were associated with an increase in 1000-day mortality and HF events in present study. These results suggest that the patients who experienced excessive mitochondrial fission during AHF time-coarse lead to adverse outcome. Mitochondrial dynamics is constantly changing by each situation. As the hemodynamics and clinical features changed dramatically within a short time after the onset of AHF, the evaluation of time-dependent changes in miRNA levels may be appropriate. Considering these, evaluation of time-dependent changes of mitochondrial dynamics during the treatment of human AHF was also novelty of present study. The present study suggests that the prognostic importance of miR-140-3p levels in the early recovery of the equilibrium of mitochondrial dynamics in patients treated for AHF. Further interventional trials of the equilibrium of mitochondrial dynamics will be required to investigate the association between the recovery of miR-140-3p levels and clinical outcomes.

The disease conditions of patients with chronic HF and those with AHF differ, as patients with AHF are hemodynamically unstable. We therefore focused on patients in whom low miR-140-3p levels continued or developed despite optimal therapy. Several explanations for the continuance and/or development of low miR-140-3p levels may be proposed. One possibility, which is our hypothesis, is that such patients are poor responders to treatment for AHF. If the AHF is not sufficiently compensated despite appropriate treatment/support, the instability in hemodynamics will continue, subsequently inducing hypoxia of the cardiomyocytes. We previously reported that high miR-140-5p levels in biopsy samples were associated with poor outcomes in patients with HF^3^. The increase in mitochondrial dynamics-related RNAs resulted in the production of reactive oxygen species (ROS) and induction of oxidative stress, which in turn induced mitochondrial fission and apoptosis. Oxidative stress adversely affects patients with HF, which explains why high miR-140-5p levels are associated with adverse outcomes in patients with hemodynamically stable HF. Thus, the sustained high levels of miR-140-5p observed in patients with chronic HF with a worse prognosis are expected. A question that arose from our study is why high miR-140-3p levels upon admission were not associated with a poor prognosis. MiR-140-3p levels may change dramatically because the hemodynamics and status of ROS of such patients often change over a short period. Even if their miR-140-3p levels are temporarily high upon admission, achieving a rapid reduction in the miR-140-3p levels will suppress the degree of oxidative stress. Thus, we hypothesize that the contrary effect of miRNA levels upon admission between patients with chronic HF and those with AHF may be a result of time-dependent changes that occur during AHF treatment.

The present study was subject to certain limitations. First, as it was a single-center study, patient-selection biases might have been included. The current sample size might appear to be small and potentially underpowered. Furthermore, there is a sample imbalance issue, with an overrepresentation of elderly participants. The current sample size and this demographic skew might affect the study’s conclusions. Second, we did not perform subgroup analysis of miRNAs according to the specific condition (e.g., type of LVEF and clinical profile) because the sample was too small. As AHF is recognized as a heterogeneous disease, subgroup analysis will be required in future studies. Third, we have no statistical evidence of grouping criteria for low value of miR-140-3p. The cut-off value was not proposed in previous reports, we therefore used Q1 to extract the patients who had extremely low miR-140-3p value. Further statistical evidence would be needed for decision of cut-off value. Forth, we could not evaluate association between miRNAs and other factor. Some comorbidities (e.g. diabetes and renal function) might affect the value of miRNAs. Finally, we could not show the prognostic impact of time-dependent changes of miRNAs. Future study will be recommended by the grouping which reflect initial status versus longitudinal changes.

## Conclusions

MiR-140-3p and miR-30-3p levels continued to decrease during treatment for AHF. Having low miR-140-3p levels during at least two of three time points despite appropriate treatment was an independent risk factor for both all-cause mortality and HF events. These results underscore the prognostic importance of severe suppression mitochondrial fission during AHF treatment.

## Data Availability

The authors confirm that the data supporting the findings of this study are available within the article. Raw data that support the findings of this study are available from the corresponding authors on reasonable request.

## References

[1] Zhao, Y. et al. MiR-485-5p modulates mitochondrial fission through targeting mitochondrial anchored protein ligase in cardiac hypertrophy. *Biochim. Biophys. Acta Mol. Basis Dis.* **1863**, 2871–2881 (2017). 28782654 10.1016/j.bbadis.2017.07.034

[2] Gu, D. et al. Mesenchymal stromal cells derived extracellular vesicles ameliorate acute renal ischemia reperfusion injury by inhibition of mitochondrial fission through miR-30. *Stem Cells Int* **2016**, 2093940 (2016). 27799943 10.1155/2016/2093940 PMC5069372

[3] Shirakabe, A. et al. Prognostic impact of excessive mitochondrial fission in patients with heart failure and evaluation of mitochondrial dynamics-related miRNAs in heart failure. *Hypertens. Res.*10.1038/s41440-025-02338-1 (2025) (**in press**). 40903535 10.1038/s41440-025-02338-1

[4] Okazaki, H. et al. Time-dependent changes in plasma xanthine oxidoreductase during hospitalization of acute heart failure. *ESC Heart Fail* **8**, 595–604 (2021). 33300276 10.1002/ehf2.13129 PMC7835601

[5] Ponikowski, P. et al. 2016 ESC Guidelines for the diagnosis and treatment of acute and chronic heart failure: The Task Force for the diagnosis and treatment of acute and chronic heart failure of the European Society of Cardiology (ESC). Developed with the special contribution of the Heart Failure Association (HFA) of the ESC. *EurJ Heart Fail* **18**, 891–975 (2016). 27207191 10.1002/ejhf.592

[6] Tsutsui, H. et al. JCS/JHFS 2021 guideline focused update on diagnosis and treatment of acute and chronic heart failure. *Circ. J.* **85**, 2252–2291 (2021). 34588392 10.1253/circj.CJ-21-0431

[7] Li, J. et al. Mitofusin 1 is negatively regulated by microRNA 140 in cardiomyocyte apoptosis. *Mol Cell Biol* **34**, 1788–1799 (2014). 24615014 10.1128/MCB.00774-13 PMC4019028

[8] Iwatani, N. et al. Different characteristics of mitochondrial dynamics-related miRNAs on the hemodynamics of pulmonary artery hypertension and chronic thromboembolic pulmonary hypertension. *J. Cardiol.* **78**, 24–30 (2021). 33836917 10.1016/j.jjcc.2021.03.008

[9] Uchikado, Y., Ikeda, Y. & Ohishi, M. Current understanding of the pivotal role of mitochondrial dynamics in cardiovascular diseases and senescence. *Front. Cardiovasc. Med.* **9**, 905072 (2022). 35665261 10.3389/fcvm.2022.905072 PMC9157625

[10] Ikeda, Y. et al. Endogenous Drp1 mediates mitochondrial autophagy and protects the heart against energy stress. *Circ. Res.* **116**, 264–278 (2015). 25332205 10.1161/CIRCRESAHA.116.303356

[11] Ikeda, Y. et al. Molecular mechanisms mediating mitochondrial dynamics and mitophagy and their functional roles in the cardiovascular system. *J. Mol. Cell Cardiol.* **78**, 116–122 (2015). 25305175 10.1016/j.yjmcc.2014.09.019 PMC4268018

[12] Shirakabe, A. et al. Drp1-dependent mitochondrial autophagy plays a protective role against pressure overload-induced mitochondrial dysfunction and heart failure. *Circulation* **133**, 1249–1263 (2016). 26915633 10.1161/CIRCULATIONAHA.115.020502 PMC4811679

[13] Chen, H. et al. Titration of mitochondrial fusion rescues Mff-deficient cardiomyopathy. *J. Cell Biol.* **211**, 795–805 (2015). 26598616 10.1083/jcb.201507035 PMC4657172

[14] Li, X. et al. Plasma miR-122 and miR-3149 potentially novel biomarkers for acute coronary syndrome. *PLoS ONE* **10**, e0125430 (2015). 25933289 10.1371/journal.pone.0125430 PMC4416808

[15] Karakas, M. et al. Circulating microRNAs strongly predict cardiovascular death in patients with coronary artery disease-results from the large AtheroGene study. *Eur. Heart J.* **38**, 516–523 (2017). 27357355 10.1093/eurheartj/ehw250

[16] Sakata, Y. & Shimokawa, H. Epidemiology of heart failure in Asia. *Circ. J.* **77**, 2209–2217 (2013). 23955345 10.1253/circj.cj-13-0971

[17] Shimokawa, H., Miura, M., Nochioka, K. & Sakata, Y. Heart failure as a general pandemic in Asia. *Eur. J. Heart Fail* **17**, 884–892 (2015). 26222508 10.1002/ejhf.319

[18] Shirakabe, A. et al. Clinical significance of matrix metalloproteinase (MMP)-2 in patients with acute heart failure. *Int. Heart J.* **51**, 404–410 (2010). 21173516 10.1536/ihj.51.404

[19] Shirakabe, A. et al. Prognostic impact of acute kidney injury in patients with acute decompensated heart failure. *Circ. J.* **77**, 687–696 (2013). 23207958 10.1253/circj.cj-12-0994

[20] Shirakabe, A. et al. Type III procollagen peptide level can indicate liver dysfunction associated with volume overload in acute heart failure. *ESC Heart Fail* **9**, 1832–1843 (2022). 35289118 10.1002/ehf2.13878 PMC9065836

[21] Sawatani, T. et al. Clinical significance of the N-terminal pro-brain natriuretic peptide and B-type natriuretic peptide ratio in the acute phase of acute heart failure. *Eur. Heart J. Acute Cardiovasc. Care* **10**, 1016–1026 (2021). 34432003 10.1093/ehjacc/zuab068

[22] Nishigoori, S. et al. Trends in sudden death following admission for acute heart failure. *Am. J. Cardiol.* **178**, 89–96 (2022). 35831216 10.1016/j.amjcard.2022.05.024

[23] Kiuchi, K. et al. The prognostic impact of hospital transfer after admission due to acute heart failure. *Int. Heart J.* **62**, 1310–1319 (2021). 34853224 10.1536/ihj.21-126

[24] Shirakabe, A. et al. Trends in the management of acute heart failure requiring intensive care. *Am. J. Cardiol.* **124**, 1076–1084 (2019). 31383351 10.1016/j.amjcard.2019.06.025

[25] Matsushita, M. et al. Social determinants are crucial factors in the long-term prognosis of severely decompensated acute heart failure in patients over 75 years of age. *J. Cardiol.* **72**, 140–148 (2018). 29523453 10.1016/j.jjcc.2018.01.014

[26] Nozaki, A. et al. The prognostic impact of gender in patients with acute heart failure—An evaluation of the age of female patients with severely decompensated acute heart failure. *J. Cardiol.* **70**, 255–262 (2017). 28040396 10.1016/j.jjcc.2016.11.015

[27] Pang, P. S. et al. Editor’s choice-the role of the emergency department in the management of acute heart failure: An international perspective on education and research. *Eur. Heart J. Acute Cardiovasc. Care* **6**, 421–429 (2017). 26265736 10.1177/2048872615600096

[28] Takahashi M, Kohsaka S, Miyata H, Yoshikawa T, Takagi A, Harada K, et al. Tokyo CCUNC. Association between prehospital time interval and short-term outcome in acute heart failure patients. *J. Card Fail* **17**, 742–747 (2011). 10.1016/j.cardfail.2011.05.005 21872144

